# Early Damage Detection in Composites during Fabrication and Mechanical Testing

**DOI:** 10.3390/ma10070685

**Published:** 2017-06-22

**Authors:** Neha Chandarana, Daniel Martinez Sanchez, Constantinos Soutis, Matthieu Gresil

**Affiliations:** 1 I-Composites Lab, School of Materials, University of Manchester, Manchester M1 3NJ, UK; dan2mar2san@gmail.com; 2 Aerospace Research Institute, University of Manchester, Manchester M1 3NJ, UK; constantinos.soutis@manchester.ac.uk

**Keywords:** acoustic emission, composites, distributed strain, optical fibre, piezoelectric sensors, structural health monitoring

## Abstract

Fully integrated monitoring systems have shown promise in improving confidence in composite materials while reducing lifecycle costs. A distributed optical fibre sensor is embedded in a fibre reinforced composite laminate, to give three sensing regions at different levels through-the-thickness of the plate. This study follows the resin infusion process during fabrication of the composite, monitoring the development of strain in-situ and in real time, and to gain better understanding of the resin rheology during curing. Piezoelectric wafer active sensors and electrical strain gauges are bonded to the plate after fabrication. This is followed by progressive loading/unloading cycles of mechanical four point bending. The strain values obtained from the optical fibre are in good agreement with strain data collected by surface mounted strain gauges, while the sensing regions clearly indicate the development of compressive, neutral, and tensile strain. Acoustic emission event detection suggests the formation of matrix (resin) cracks, with measured damage event amplitudes in agreement with values reported in published literature on the subject. The Felicity ratio for each subsequent loading cycle is calculated to track the progression of damage in the material. The methodology developed here can be used to follow the full life cycle of a composite structure, from manufacture to end-of-life.

## 1. Introduction

Fibre reinforced polymer (FRP) composites are known to be stronger, more corrosion resistant, and more lightweight than traditional metals and metal alloys. Improvements in the achievable properties and manufacturing techniques has led to an increase in their use in critically loaded structural applications. However, with new techniques comes uncertainty of the parameters involved. It is not uncommon to find voids, dry and resin rich regions, and other imperfections in finished composite parts. Defects and damage can often be imparted during fabrication. These and the inherent nature of the process can lead to residual strains in the finished product, which may affect the overall performance. Non-destructive inspection/evaluation (NDI/E) techniques are often employed for the detection, localisation, and quantification of flaws and damage. Widely researched methods include the use of X-rays [1], ultrasonic waves [2], eddy currents [3], shearography [4,5,6], and infrared thermography [7,8,9,10]. Though they improve safety and often minimise premature replacements, inspections can represent significant down-time and labour costs. In the aerospace industry, for example, inspection of composite structures accounts for one third of the operation costs [11], so it becomes necessary to develop in-situ monitoring methods. Structural health monitoring (SHM) systems are permanently integrated within structures using embedded or surface mounted sensor networks [12]. The layered nature of FRP composites allows small sensors to be integrated, thus allowing real time process monitoring. Optical fibres [13,14,15] and piezoelectric sensors [12,16,17,18,19,20] have been introduced for SHM in composite materials. 

This paper presents an experimental study where a distributed optical fibre sensor is embedded and used to follow the full manufacturing process of a composite laminate, providing valuable information related to the rheology of the system and captures the magnitude of residual strains. During mechanical four point bending, distributed strain and acoustic emissions are monitored to be able to detect the initiation and monitor the progress of damage in the structure as the maximum load is increased. The use of these ‘smart sensors’ has proven to be effective and reliable in the authors’ and others’ previous work when applied to the SHM of composite materials. Similar experimental observations are made in this work, thus validating that it is scientifically sound and has relevance in this research field.

## 2. Distributed Optical Fibre Sensors

Introduced in the late 80s to early 90s, fibre Bragg grating (FBG) sensors are the most common optical fibre sensor in SHM applications [14,21]. FBGs comprise a grating—periodically modulated refractive index—along a segment of optical fibre which acts as an optical filter by reflecting the Bragg wavelength while transmitting others. The spacing of the grating is a function of strain and temperature, so when the sensor experiences mechanical and/or thermal strains, the Bragg wavelength undergoes a shift. FBG sensors have been employed in many cases for in-situ and real time monitoring of composites due to their non-invasive nature [21,22,23,24], but, without multiplexing technology, each FBG requires its own interrogator system.

A distributed optical fibre sensor (DOFS) is a single optical fibre, sensitive at multiple points along its length [25]; thus, it is possible to use a single distributed sensor in the place of several discrete sensors. When a narrow band of light is passed through the core of a DOFS, three different scattering processes take place: Raman, Brillouin, and Rayleigh scattering [26]. The higher signal intensity and losses below 20 dB/km from Rayleigh-based systems makes them favourable [27]. Rayleigh scattering occurs due to interactions between the light and silica glass core; this allows individual sensors to be distinguished by their unique “fingerprint”. Optical time-domain reflectometry (OTDR) is a known technique for distributed sensing using Brillouin or Raman scattering, but the sensing range is limited by the reliance on signal intensity by OTDR systems. Improved spatial resolution can be achieved by optical frequency domain reflectometry (OFDR). OFDR systems use a variable frequency laser beam coupled into an interferometer and are capable of providing measurements with high spatial resolution (~1 mm) and the potential to resolve strain and temperature measurements as fine as 1 µstrain and 0.1 °C, respectively [28]. The mechanical effect of embedding a single-mode, low-bend-loss optical fibre (~155 µm diameter) has been shown to be negligible [29], making distributed sensing with optical fibres one of the few viable methods for process monitoring as well as in-service monitoring.

## 3. Acoustic Emission in Composite Materials

**Figure 1:**
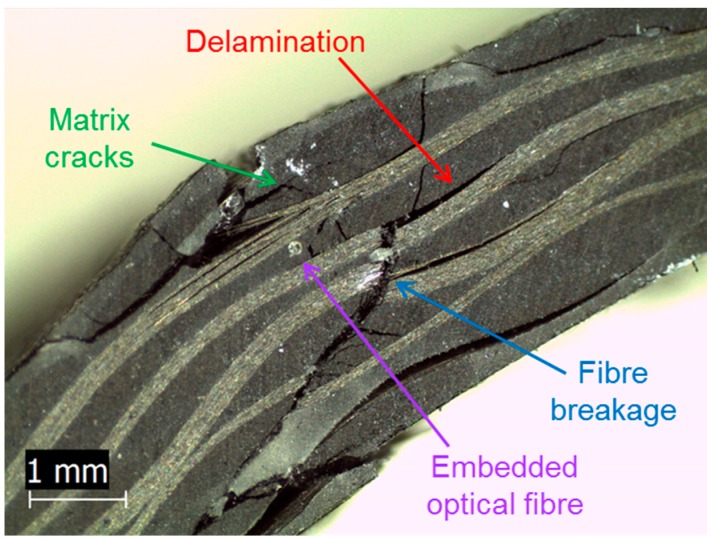
Cross-section of damaged carbon fibre woven composite with embedded optical fibre.

**Figure 2:**
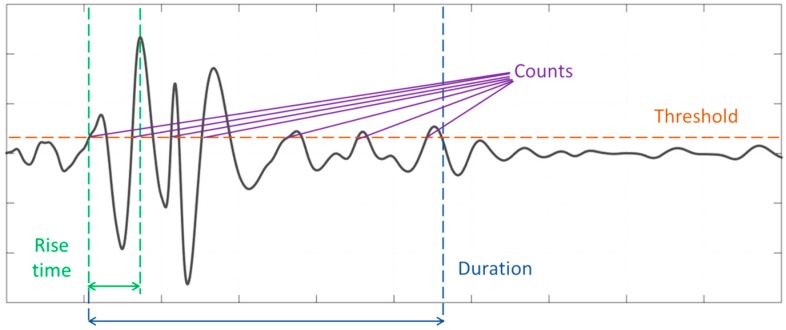
Typical acoustic emission waveform annotated with signal features.

Acoustic emission (AE) is an SHM technique which uses piezoelectric sensors as receivers of elastic waves, generated by microstructural damage, as they propagate through the material. A network of three or more sensors can be used to determine the damage source location and estimate the severity and type of damage. The use of AE for early damage detection in composites is well established [30], with the last forty years of research concluding that there are four main damage mechanisms in composite materials identifiable by their AE “signature” [31,32]: (i) matrix cracking; (ii) interfacial debonding; (iii) fibre-matrix friction/fibre pull-out; and (iv) fibre breakage. Some of these are shown in Figure 1. Researchers have used many different methods to distinguish these damage mechanisms during mechanical testing, including, but not limited to, classification of acoustic signature [16,32], amplitude and frequency distribution analysis [16,32,33,34,35,36,37,38], and analysis of different features of the waveform [32,39]. Figure 2 shows a typical AE signal and the parameters that are commonly used for analysis of AE-generating damage events. Typical waveforms have also been associated with different damage modes based on the signal geometry: A-type signals (slow increase times around 10–20 µs) are associated with matrix cracking, B-type signals (sharp rising, lasting for around 10 µs and abruptly decreasing) are associated with fibre/matrix debonding, C-type signals (very sharp rising and short duration, <10 µs) are associated with fibre breakage, and D-type signals (long rising times, high amplitudes, and long duration) are associated with delamination [32].

Table 1 and Table 2 show a compilation of the amplitude and frequency distributions used to classify different damage mechanisms from the literature. These studies clearly demonstrate the difficulty of identifying damage modes in composite materials, though there is a clear trend that shows an increase in both amplitude and frequency as the type of damage becomes more severe. The ability to record signals with an amplitude as low as 30–40 dB is made possible by use of a low signal threshold, which applied in a real structural health monitoring application would not be appropriate. In the present work, we have utilised a higher threshold value to better represent the real application of the technique. The waveforms obtained can be dependent on many parameters, including, but not limited to: the choice of sensors, method of coupling/bonding between the sensors and host structure, structural materials used, number of sensors, size of the specimen, and the arrangement and spacing of sensors.

### 3.1. Felicity Effect

When a homogeneous, isotropic specimen is loaded incrementally, a phenomenon known as the ‘Kaiser Effect’ is observed; AE signals are only emitted in subsequent loadings when the previous maximum load is exceeded. In composite materials, a similar effect is observed; this is known as the Felicity effect [55]. In composites, the friction between the constituents during loading can cause significant acoustic emission signals, without exceeding the previous maximum load. A Felicity ratio can be calculated as:

$$\mathrm{Felicity}\;\mathrm{Ratio},\;\mathrm{FR}=\;\frac{\mathrm{Load}\;\mathrm{when}\;\mathrm{AE}\;\mathrm{resumes}}{\mathrm{Maximum}\;\mathrm{applied}\;\mathrm{load}}$$

During subsequent loadings of a component, a reduction in the Felicity ratio generally tends to be observed. Observation of the load at which AE events start to occur during a mechanical loading test can, therefore, reveal information about the load-time history of a component, providing the material properties are known.

## 4. Materials and Methods

**Figure 3:**
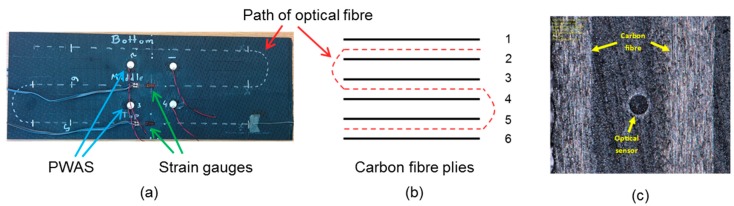
(**a**) Network of sensors in smart composite laminate; (**b**) Schematic of the cross-section showing the path of the optical fibre through-the-thickness; (**c**) Micrograph of embedded optical fibre of 155 µm diameter. PWAS = piezoelectric wafer active sensors.

A composite laminate (400 mm × 200 mm) was manufactured by vacuum assisted resin infusion moulding of six plies of carbon fibre fabric—5-harness satin weave, supplied by Cytec Solvay Group (formerly ACG, NJ, USA)—with an epoxy resin system (Araldite LY 564 and Aradur 2594, supplied by Huntsman, Basel, Switzerland). For more information about the manufacturing process, the reader is referred to [56]. A single-mode, low-bend-loss, polyimide coated silica glass optical fibre sensor, of 155 µm diameter and 2 m length, was embedded during the lay-up process to give three defined strain sensing regions near the ‘top’, ‘middle’, and ‘bottom’ surface of the laminate in the panel (Figure 3a). The distance between each parallel sensing region was 50 mm. Figure 3b is a schematic of the cross-section illustrating the path of the optical fibre through the thickness of the panel. Strain data was recorded from the optical fibre during resin infusion and curing. Three K-type thermocouples were embedded in the panel for measurement of temperature during the curing process. Each thermocouple was embedded at the same position through-the-thickness as the three optical fibre sensing regions. For AE monitoring, piezoelectric wafer active sensors (PWAS)—PIC255 with 10 mm diameter and 0.5 mm thickness [57]—were bonded to the surface of the panel using a cyanoacrylate adhesive.

After manufacture, quasi-static four point bending was conducted on the plate (trimmed to 385 mm × 145 mm) using an adaptation of the ASTM standard D7264: ‘Flexural properties of polymer matrix composite materials’ on an Instron 5969 testing machine fitted with a 10 kN load cell (Figure 3). Though more complex than uniaxial loading, four point bending offers the ability to observe tensile and compressive strains contained within a single specimen. Aluminium ‘spreader bars’ are placed between the specimen and loading noses to ensure even distribution of the load across the width of the panel. In the initial stages of this work, five progressive quasi-static loading steps were completed. Loading steps 6–13 were completed at a later date for validation of each measurement method. The maximum strain observed in each of the cycles is stated in Table 3. 

**Figure 4:**
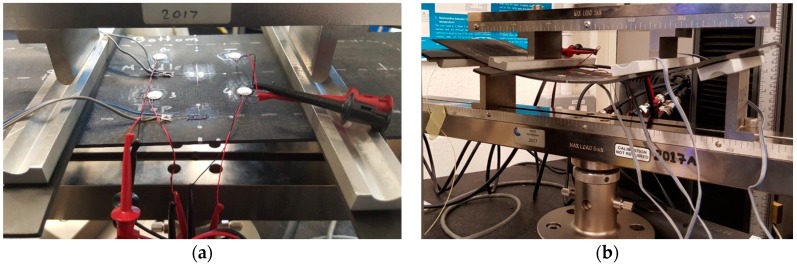
Four point bending of the laminate (**a**) before loading; (**b**) during loading.

During each loading, the surface strain on the top and bottom surfaces of the panel was measured by uniaxial strain gauges. Four piezoelectric wafer active sensors (PWAS) acted as receivers of AE during loading were bonded on the top of the plate (Figure 4a). Figure 4b shows the mechanical four point bending set-up. Distributed strain data was collected from the optical fibre sensor via an optical frequency domain reflectometry (OFDR) based interrogator (ODiSI-B model) from Luna Inc. (Roanoke, VA, USA) while AE data was recorded by the software ‘AEWin for PCI2’ (version E5.60) from Mistras Group (NJ, USA) with a sampling rate of 10 MHz and 20 dB of pre-amplification per sensor. A threshold amplitude value (58–65 dB) was assigned to each PWAS based on its sensitivity to extraneous noise during preliminary pencil-lead-break tests. 

## 5. Results and Discussion

### 5.1. Manufacturing Process Monitoring

**Figure 5:**
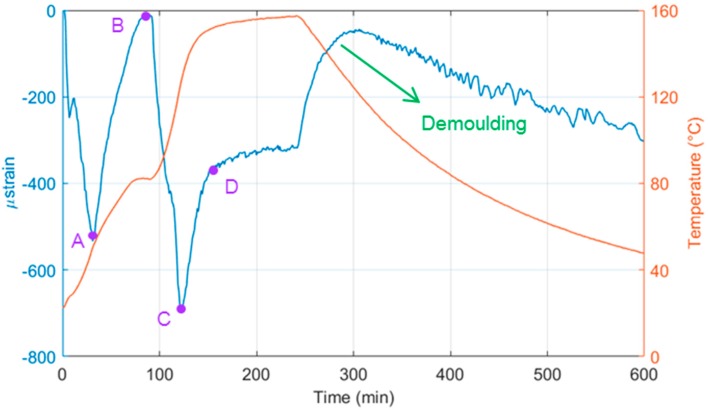
Temperature measured by thermocouples (**red**) and mechanical strain measured by the embedded optical fibre (**blue**) in the panel during the curing cycle of the composite laminate.

Resin infusion was carried out at room temperature, using a vacuum to draw the resin through the fibres. During this process, the strain in the optical fibre can be attributed only to mechanical strain. During the curing process, thermocouples were used to follow the change in temperature, while the DOFS measures the total strain, which comprises two components: mechanical strain and thermal strain. The temperature influences greatly the total strain in the panel, thus concealing the mechanical strain that results from the curing process; so, using data obtained from a short length of optical fibre, sensitive only to changes in temperature, it was possible to offset the total strain to give the value of mechanical strain. When plotted against temperature, taken as an average from the three thermocouples, the effect of each stage of the curing cycle on the mechanical strain experienced by different regions of the laminate becomes clear (Figure 5). During the first ramp in temperature to 80 °C (1 °C/min) the resin begins to contract as its viscosity decreases, causing a sharp increase in compressive mechanical strain. This increase continues until the first stationary point in mechanical strain (A). The continuing increase in temperature forces thermal expansion of the composite—and reduction in strain—until no additional energy is made available to aid this process, thus, the dwell point (B) is reached. A second ramp in temperature to 160 °C causes a second increase in compressive strain; the additional thermal energy aids further crosslinking between the resin components until the last turning point (C), when the resin goes through a glass transition. A decrease in chemical contraction sees a decrease in compressive strain until point D, after which the rate of chemical contraction reaches a plateau. During cooling, contraction of the panel causes, once again, an increase in mechanical strain.

**Figure 6:**
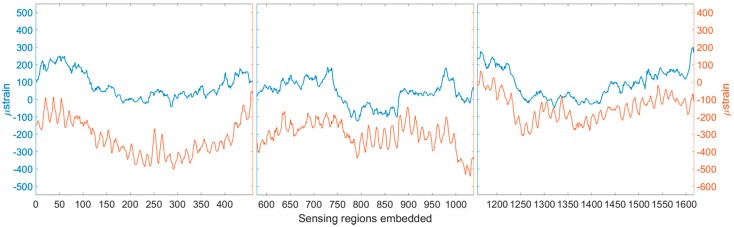
Strain developed in the panel during resin infusion (**blue**) and the residual strain in the panel after manufacture (**red**). Left to right: top, middle, and bottom optical fibre sensing regions.

Figure 6 shows the residual strain profile of the optical fibre in the finished composite panel. When compared with the strain developed in the panel during the resin infusion process, there is a clear correlation. This observation can be particularly helpful when manufacturing complex composite parts, since it becomes possible to predict the residual strain profile of the finished part before completing the full manufacturing process. Residual strains could impact the damage tolerance of the final part and are, therefore, of interest in many applications. The detection of manufacturing defects such as resin rich areas or dry fibres has also been possible using distributed optical fibres in previous work by the authors [13].

### 5.2. Four Point Bending Monitoring

#### 5.2.1. Distributed Strain Monitoring

**Figure 7:**
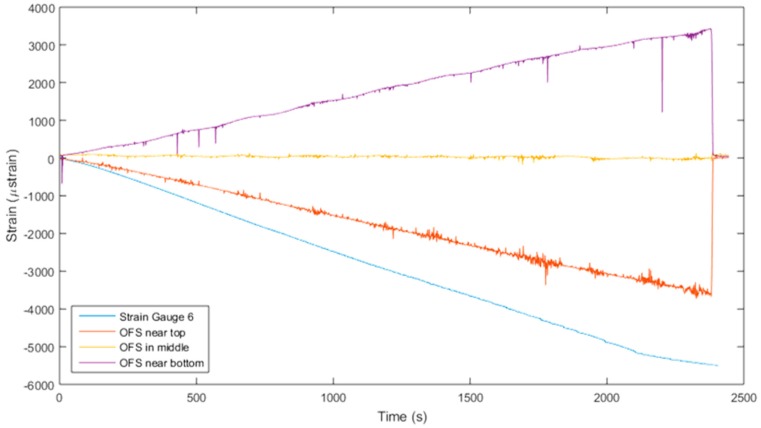
Development of surface strain measured by SG6 and strain in the optical fibre during bending cycle 13.

Figure 7 shows the development of strain in the panel during the final loading step as recorded by both the optical fibre and the surface mounted strain gauges. The ‘middle’ sensing region, embedded along the neutral axis of the cross-section of the laminate, successfully records a neutral strain reading during loading. A good correlation is observed between the strain gauges and optical fibre strain, with lower values of strain recorded by the optical fibre attributed to the respective positioning of each sensing region through-the-thickness of the laminate. The thickness of the composite panel is approximately 3 mm, so the three sensing regions in the optical fibre can be approximated to be positioned at 0.5 mm, 1.5 mm, and 2.5 mm through-the-thickness. Since the normal stress of the longitudinally oriented optical fibre is proportional to its distance from the neutral surface, the sensitivity of the placement of the optical fibre through-the-thickness is perfectly demonstrated here. 

**Figure 8:**
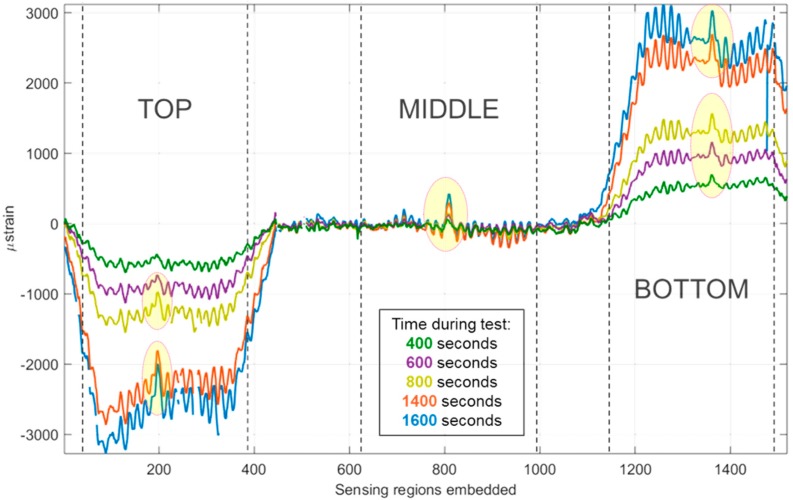
Strain developed in the panel during cycles 1–5.

Figure 8 shows the strain developed in the optical fibre during each loading of the plate, up to a maximum measured strain of 0.4% on the central strain gauge. Once again, the sensitivity of the optical fibre sensor (OFS) through-the-thickness of the plate is clearly demonstrated by the compressive, neutral, and tensile strains recorded by the ‘top’, ‘middle’, and ‘bottom’ sensing regions, respectively.

**Figure 9:**
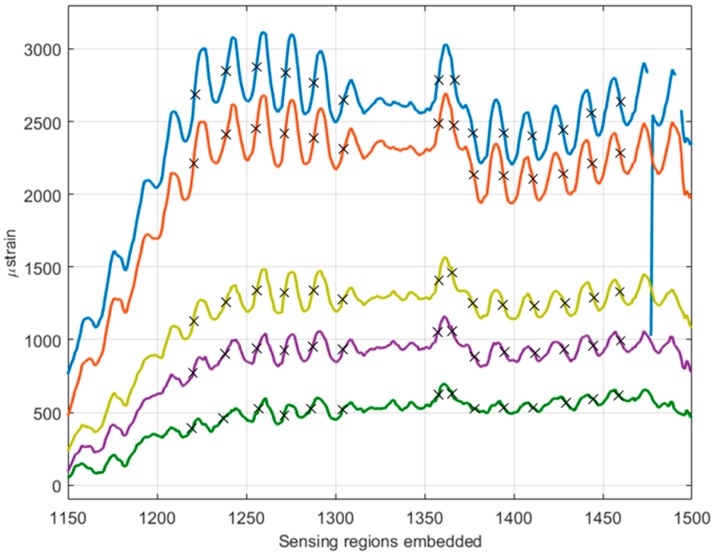
Strain developed in the ‘bottom’ sensing region during cycles 1–5 with wavelength measurement points annotated.

A small peak is visible in each measurement region, increasing in amplitude from 400 s to 1600 s. On first glance it may appear that this is a concentration of stress caused by the presence of local damage in the structure which occurred during loading. A more plausible possibility is that this peak is caused by yarn crimp within the fabric plies above and below the embedded optical fibre sensor. With this in mind, the possible nesting characteristics of the fabric plies above and below the optical fibre were investigated. Figure 9 shows clearly the oscillating nature of the distributed strain signal at different stages of the experiment. The wavelength of the peaks seen in the signal was calculated as an average from multiple peaks in the bottom and top sensing regions; this data is displayed in Table 4. Each data point is 0.625 mm away from the next, allowing the wavelength to be calculated in millimetres. The values from different sensing regions are consistent, confirming that the effect of geometry at different thickness levels is similar. A model of two fabric layers was produced in the open source software, TexGen (v3.9.0, Nottingham, UK) [58]. 

**Figure 10:**
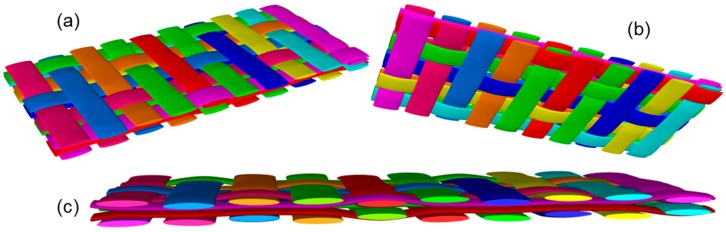
TexGen model showing the possible nesting of two layers of 5-harness satin fabric: (**a**) top view; (**b**) bottom view; (**c**) view of the cross-section.

**Figure 11:**
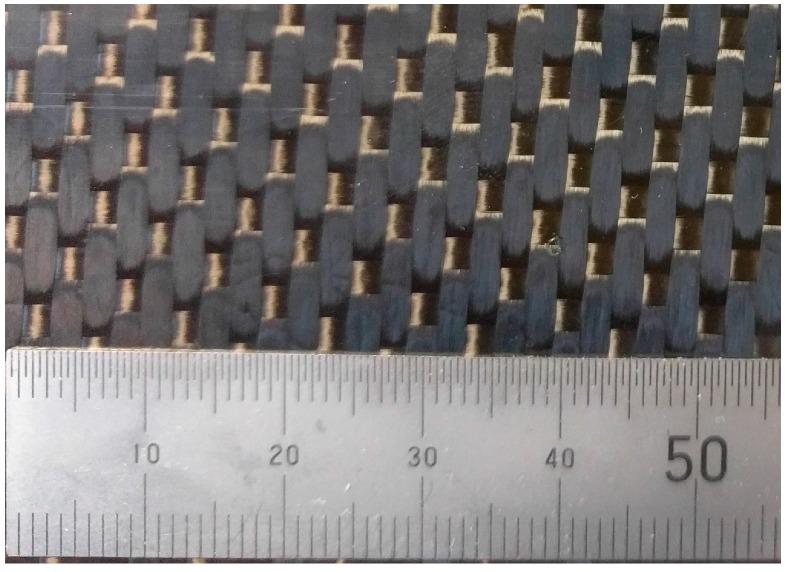
Photograph of the laminate showing the 5-harness satin weave. Repeat size ~10.1 mm.

Figure 10 shows the potential nesting of two layers. Figure 11 is a photograph of the laminate used in this work, showing clearly that the weave repeats in the orientation of the optical fibre approximately every 10.1 mm. This value correlates well with the measurements from optical fibre data (i.e., ~10.45 mm), thus supporting the hypothesis that the geometry of the plot is a consequence of the fabric nesting which results in local strain variation.

#### 5.2.2. Acoustic Emission Monitoring

**Figure 12:**
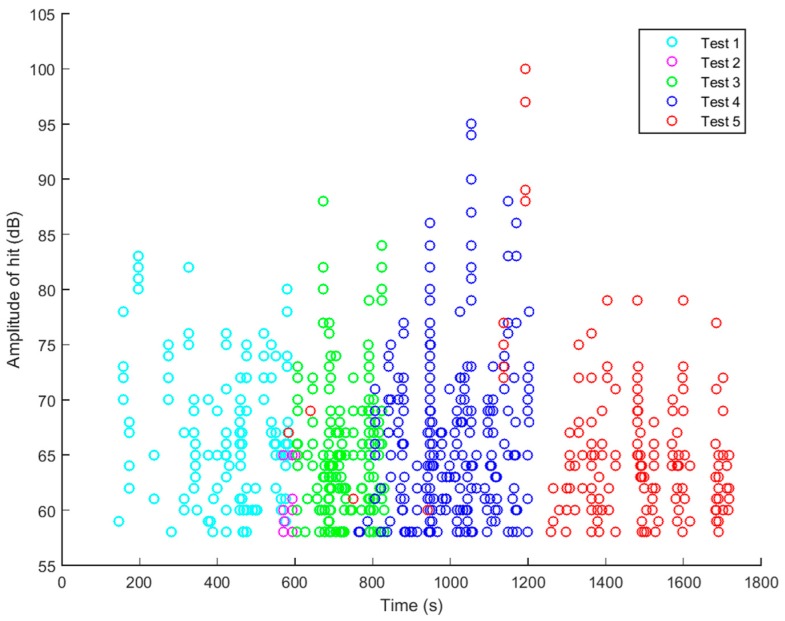
Maximum amplitude of acoustic emission hits above the threshold gain received by all piezoelectric sensors during cycles 1–5.

Figure 12 is a plot of the maximum amplitude of each AE ‘hit’ above the threshold gain recorded from all of the PWAS sensors during loadings 1–5. The threshold for each PWAS was set between 58–65 dB and digital filtering (100 kHz–1 MHz) was used to eliminate extraneous noise in the acoustic range. When plotted on a single graph, it becomes clear that the majority of AE hits during each loading are recorded mainly after exceeding the previous maximum load. Based on this, the Felicity ratio of each loading was calculated using the equation presented in Section 3.1; the results are shown in Table 5. It is seen here that on increasing the loading of the specimen, the felicity ratio decreases; this is to say that the number of AE events recorded prior to reaching the previous maximum load/extension increases as the load is increased in each cycle. The data presented here fits with the theory of the Felicity effect.

**Figure 13:**
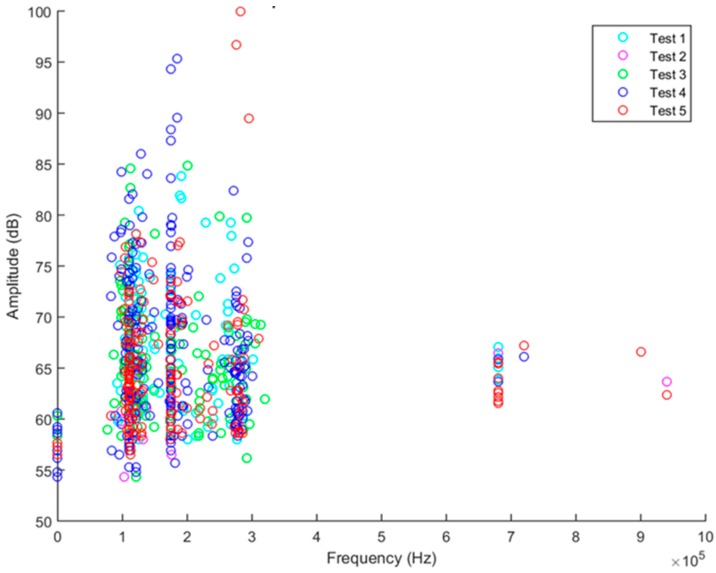
Maximum amplitude versus peak frequency of acoustic emission hits received during cycles 1–5.

If analysis of the recorded acoustic emission signals was constrained to the distribution of amplitude, as many researchers have done in the past, the AE signals recorded would suggest the formation of matrix cracks. The general trend for AE hit amplitudes is to increase as the strain is increased, demonstrating the development of damage in the structure. Particularly high amplitude hits in the range 90–100 dB, which researchers [33,35,44,46] have previously attributed to fibre fracture, are observed during the fourth and fifth loading steps of the plate. The data could, in fact, be interpreted to show micro fibre breakage observed due to stress concentration at the tip of a crack or delamination, as mentioned by Gresil et al. [16], since the signals are received at relatively low levels of strain where fibre fracture would not be expected to occur. However, analysis of the maximum amplitude-peak frequency distribution during the same loading cycles (Figure 13) reveals that there are very few high frequency events (700 kHz–1 MHz) which researchers have previously attributed to high energy damage mechanisms. The majority of the AE activity is focused around 100–300 kHz during all of the cycles. In addition, the high amplitude hits previously encountered fall into this low frequency range. It is possible to conclude from this, with confidence, that the high amplitude is not related to the size of the damage event, but perhaps is a consequence of sensor position. 

**Figure 14:**
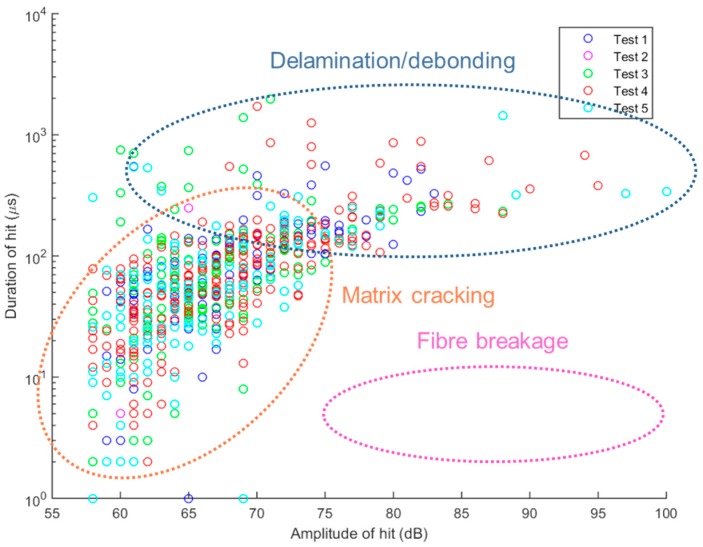
Duration of received signals versus maximum amplitude during cycles 1–5.

**Figure 15:**
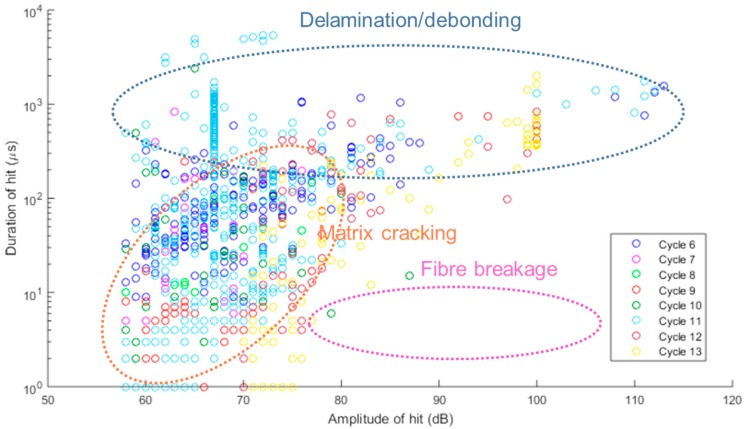
Duration of received signals versus maximum amplitude during cycles 6–13.

Figure 14 and Figure 15 show the relationship between signal duration and maximum amplitude of acoustic emission signals received during cycles 1–5 and 6–13, respectively. The trend is in agreement with work presented by Kotsikos et al. [39]. Taking into account the different signal types described by researchers [16,32] in the past, we have been able to speculate with a higher degree of certainty, on the damage types exhibited here: A-type signals—associated with matrix cracking—tend towards the lower amplitudes while signal durations can vary; C-type signals are short signals (~10 μs duration) with high amplitudes, associated with fibre breakage; D-type signals with high amplitudes and long duration are associated with delamination [32]. These figures further support the suggestion that the cycles of loading and unloading on this specimen have not caused severe damage to the material. Further work is required on analysis of signals based on sensor type, sensor location, bonding layer properties, etc. to be able to decipher the results with more confidence.

## 6. Concluding Remarks

This work has shown the ability to combine two different types of sensors for structural health monitoring of composite structures. Distributed strain monitoring during fabrication can provide valuable information about the resin rheology in addition to mechanical properties of a part prior to the completion of its manufacture. Once it goes into service, the embedded optical fibre can continuously monitor applied strains that could determine its performance. The undulating geometry of strain data recorded by the optical fibre sensor is a result of crimp in the yarns which make up the layers of fabric above and below the optical fibre sensing regions. These small peaks are accentuated as the strain is increased—an observation which should be taken into account when this technique is used in service.

The use of AE monitoring has shown promise in the early distinction and quantification of damage mechanisms in composite materials. The Felicity effect can be used to determine the time-load history of a component. When combined with historical strain data, this information can be particularly beneficial in informing the design of structures based on the distribution and concentration of strains. The importance of taking into account multiple features in each AE signal to be able to distinguish between different damage mechanisms has been highlighted in this work. In addition, the material/sensor system should not be neglected in the interpretation of the results. To use acoustic emission monitoring in-service, it is important to consider the effect of extraneous noise on the system and to define carefully an appropriate threshold. It is possible to use the PWAS to calculate an AE source location based on the time of arrival of signals to different sensors. Repetition of damage in the same or different locations can, therefore, be distinguished and correlated against any residual strains observed along the length of the embedded optical fibre. In near future work, AE data will be validated by sectioning and optical microscopy as well as by conventional non-destructive testing (NDT) techniques. 

## Figures and Tables

**Table 1:** Amplitude distribution of acoustic emission (AE) signals in composites. (CF = carbon fibre; CFRP = CF reinforced polymer; Cy = cyclic; Ep = epoxy; GF = glass fibre; PET = polyester; PP = polypropylene; Qs = quasi-static; 3PB/4PB = 3/4 point bending; Tens. = tensile).

Ref.	Material	Test	Sensor	Amplitude Distribution (dB)			
				Matrix Cracking	Interfacial Debonding	Fibre/Matrix Friction and Fibre Pull-Out	Fibre Breakage
[40]	-	-	-	30–45	45–55	-	>55
[41]	Graphite/Glass	Tens., 4PB	PAC pico	60–80	70–90	-	-
[42]	CF/Ep	Buckling, 3PB	R15	43–65	45–75	50–85	-
[43]	CFRP	Tens. Cy	Fuji ceramic M204	40–70	-	-	60–100
[39]	GF/PET	Immersed bending fatigue	PAC U30D03	40–60	60–80	-	80–100
[44]	GF/Ep	Torque	WD AE	32–72	46–68	69–86	87–100
[45]	GF/Ep	3PB	PZT disc	35–82	50–95	-	>65
[33]	GF sandwich	3PB static/fatigue	PZT disc	40–76	72–100	-	>94
[46]	GF/PP	Tens., crack propagation	PAC micro 80	40–55	60–65	65–85	85–95
[32]	GF/Ep	Tens.	PAC micro 80	40–80	50–80	70–100	-
[47]	CF/Ep	Tens.	PAC pico	<70	<60	-	-
[34]	CF/Ep	Tens.	Digital wave B1025	35–55	55–100	-	35–80
[35]	CF/Ep notched	Tens.	-	50–60	60–70	-	70–100
[16]	GF/Ep	Tens.	Steminc SM412	60–100	-	-	-
[48]	GF/Ep	Ring tens.	WD AE	40–80	-	80–90	90–94
[49]	GF/Vinyl-ester	Tens.	-	45–55	55–75	-	~83

**Table 2:** Frequency distribution of AE signals in composites. (CF = carbon fibre; CFRP = CF reinforced polymer; Cy = cyclic; Ep = epoxy; GF = glass fibre; PET = polyester; PP = polypropylene; Qs = quasi-static; 3PB/4PB = 3/4 point bending; Tens. = tensile).

Ref.	Material	Test	Sensor	Frequency Distribution (kHz)			
				Matrix Cracking	Interfacial Debonding	Fibre/Matrix Friction and Fibre Pull-Out	Fibre Breakage
[50]	Graphite/Ep	Tens.	Panametrics 5070AE	50–150	-	-	140–180
[51]	GF/PET	-	-	30–150	30–100	180–290	300–400
[52]	GF/PET	-	-	80–130	-	250–410	250–410
[47]	CF/Ep	Tens. Qs/Cy	PAC Pico	~300	-	300	>500
[53]	CF/Ep	Tens., DCB, lap shear	PAC-WD	50–180	220–300	180–220	>300
[36]	GF/PP	Tens.	PAC-WD	90–110	-	200–300	>420
[37]	CF/Ep	CT, CC, DCB, 4PB	PAC-WD	<50	200–300	500–600	400–500
[38]	Graphite/Ep	Tens.	9223M3 Mini	~140	~300	-	~405
[54]	CF/GF/Ep	Tens.	B1025	200–600	200–350	700–1100	>1500
[34]	CF/Ep	Tens.	B1025	50–80	50–150	-	150–500
[16]	GF/Ep	Tens.	Steminc SM412	80–400	-	-	-

**Table 3:** Progressive loading cycles of four point bending. Test speed = 1 mm/min. Cycles 6–13 completed at a later date.

Loading Step	Strain ^1^ (%)	Loading Step	Strain ^1^ (%)
1	0.14	8	0.41
2	0.14	9	0.41
3	0.2	10	0.43
4	0.29	11	0.46
5	0.4	12	0.5
6	0.42	13	0.55
7	0.41		

^1^ Maximum strain as measured by the central strain gauge (SG6).

**Table 4:** Calculated wavelengths of oscillations in distributed strain data from the top and bottom sensing regions.

Time of Plot (s)	Bottom		Top	
	Mean Wavelength (mm)	Standard Deviation (mm)	Mean Wavelength (mm)	Standard Deviation (mm)
400	10.51	1.28	10.18	1.34
600	10.45	0.74	10.09	1.11
800	10.45	0.57	10.63	1.86
1400	10.40	0.42	10.63	1.38
1600	10.40	0.42	10.63	1.38

**Table 5:** Calculated Felicity ratios for four point bending cycles 1–5.

Loading Step	Strain ^1^ (%)	Load at Maximum Strain (N)	Felicity Ratio
1	0.14	358	1
2	0.14	349	1
3	0.2	516	0.98
4	0.29	741	0.78
5	0.4	1050	0.81

^1^ Maximum strain as measured by the central strain gauge (SG6).
